# Synergetic effects of plastic mulching and nitrogen application rates on grain yield, nitrogen uptake and translocation of maize planted in the Loess Plateau of China

**DOI:** 10.1038/s41598-018-32749-9

**Published:** 2018-09-25

**Authors:** Xiukang Wang, Ning Wang, Yingying Xing, Mohamed BEN El CAID

**Affiliations:** 1 College of Life Sciences, Yan’an University, Yan’an, Shaanxi 716000 China; 2 Laboratory of Biotechnology and Valorization of Natural Resources, Department of Biology, Faculty of Science, Ibn Zohr University, 8106 Agadir, Morocco

## Abstract

Nitrogen (N) fertilization potentially affects the amount of N uptake and N translocation (NT) within plants, but the synergetic effects of plastic mulching and N application rates on the grain yield (GY), N uptake and NT of maize have not been studied. A fertilization experiment with six N application rates (0, 80, 160, 240, 350 and 450 kg ha^−1^) with or without mulch was conducted in 2015 and 2016 in the Loess Plateau of China. There were significant interactions between mulch and the N fertilizer rate on the GY. Under mulch treatments, the highest GY was observed at 450 kg ha^−1^, which was 53.9%, 36.4%, 20.2%, 1.6% and 0.3% higher than those obtained with N application rates of 0, 80, 160, 240 and 350 kg ha^−1^, respectively, in 2015. The ranking of NT to grain N accumulation was leaves > sheaths and stems > ear axis > bracts. The NT efficiency (NTE) levels averaged over the different N fertilization rates under the no-mulch treatment were 5.6% and 12.9% higher than those under the plastic mulch treatment in 2015 and 2016, respectively. We conclude that an N fertilizer application rate of 240 kg ha^−1^ with mulch can achieve a relatively higher NTE, GY, WUE and NUE.

## Introduction

Maize is one of the most important grain crops in China and is a crop that is sensitive to fertilizer and water demand^1^. However, most maize cultivation is nearly universally rainfed or only receives supplementary irrigation in northwest China^2^. The Loess Plateau region of northwest China, where the annual rainfall ranges from 500 to 600 mm, is primarily characterized by maize monocropping systems^3^. However, to achieve successful and expanding agricultural production in this region, the problems of adverse weather, low-fertility soils, and scarce and unevenly distributed rainfall remain unsolved^4,5^. In general, most rainfall is distributed from June to September^6^, and in most years, droughts occur in the spring and occasionally in the summer or autumn. Seasonal drought, water shortages in the topsoil due to wind, and low soil temperature during the seedling period are all reasons for low or late maize seedling emergence during most years.

Much effort has been directed toward the conservation of soil moisture and the enhancement of topsoil temperature. Over the long term, a variety of materials have been used as mulches for dry farming areas in China, including straw, plastic, gravel and vegetative residues^7–10^. Among the tested approaches, plastic mulch cultivation has become a widely applied agricultural practice^11^. In 2010, plastic mulch was used on 51,000 km^2^ of arable land in China^12^, and specifically, it has been used on approximately 10,700 km^2^ for maize production in the Loess Plateau region of northwest China^13^. Plastic mulch is used to warm the topsoil in the spring and to increase soil water availability by reducing soil evaporation and preventing capillarity^14,15^. However, crop production requires the integration of soil temperature, moisture and fertility, and different levels of fertilizer input exert different effects on soil fertility and use efficiencies and thus on maize growth and yields^16^.

Nitrogen (N) is the primary and most limiting nutrient in stable crop production, and thus, N use efficiency (NUE) is important for both economic and environmental sustainability^17–19^. In the semiarid regions of China, the N fertilizer application rate for maize production ranges from 180 to 360 kg ha^−1^, but the potential maximum grain yield (GY) only ranges from 5284 to 6784 kg ha^−1 ^^20,21^. The average N fertilizer application rate for high-yielding maize in China was 237 kg ha^−1 ^^22^, but considering both the maximum economic benefit and environmental cost, the optimal N rate for maize in North China is 180 kg ha^−1 ^^21,23,24^. Furthermore, overapplication of N both increases environmental risks and reduces the economic efficiency of N fertilizer.

To improve the NUE of fertilizer, the N balance should be estimated to select the appropriate N fertilizer input levels^25–27^. However, this estimation is usually focused on the soil (N residue and leaching) — plant (N uptake and utilization) — air (N_2_O and NH_3_ emission) system. Grains have two sources of N: root uptake and vegetative organs^28,29^. Absorbed N is not directly transported to the grain; instead, it is first carried into vegetative organs and then translocated into the grain at a later stage^30^. The uptake of N from the soil by the vegetative organs and its transfer to the grain occur simultaneously^31^.

Leaves and stalks are the major sources of grain N, but the roots also make a small contribution^32,33^. Nevertheless, there is little information concerning the efficiency with which maize uptakes and allocates N to its organs at multiple reproductive stages with and without mulch and at different N fertilization rates in the Loess Plateau region of northwest China. In particular, the effects of the interactions between mulching and N application rates on N translocation (NT) among different organs remains unknown. The present study was designed to address the synergistic effect of plastic mulch and N fertilizer application rates on maize yield, NUE and NT among different organs in the Loess Plateau region of northwest China. Answering these questions is important to maximize the benefits of using plastic mulch technology in maize production and to provide a more scientific method for selecting the optimal N application levels to strengthen N fertilizer use and protect the soil environment.

## Materials and Methods

### Experimental site

This experiment was conducted in 2015 and 2016 in Ansai County, Shaanxi Province, China. The planting site (36°39′ N, 109°11′ E, 1109 m above sea level) has a mean annual air temperature of 8.7 °C and a mean annual precipitation of 511 mm. The soil developed from loess is classified as loessal soil. The average sand, silt and clay contents in the 0–80-cm soil profile were measured with a laser particle size analyzer (Dandong Haoyu Technology Co., Ltd), and the values were 18 ± 1.1%, 68 ± 1.1% and 12 ± 2.1% in 0–20 cm; 16 ± 1.4%, 67 ± 1.3% and 15 ± 1.7% in 20–40 cm; 14 ± 2.9%, 60 ± 3.4% and 22 ± 3.2% in 40–60 cm; and 12 ± 2.2%, 65 ± 2.3% and 20 ± 0.5% in 60–80 cm. The pH in the cultivated layer was 8.2 (1:2.5; soil: water), and the soil properties (n = 4, repeated four times for each value) of the top 80 cm are shown in Table 1.

**Table 1:** Major soil physicochemical characteristics of the experimental site were measured before the experiment.

Years	Soil depth (cm)	Bulk density (g cm^−3^)	Organic matter (g kg^−1^)	Total C (g kg^−1^)	Total N (g kg^−1^)	NO_3_^−^ −N (mg kg^−1^)	NH_4_^+^ −N (mg kg^−1^)	Avail. P (mg kg^−1^)	Avail. K (mg kg^−1^)
2015	0–30	1.20	12.63	11.10	0.71	9.83	2.80	38.7	144.6
	30–60	1.27	9.88	12.70	0.45	4.26	2.70	19.8	123.3
	60–80	1.32	5.49	10.00	0.39	3.18	2.00	16.6	114.5
2016	0–30	1.25	13.20	10.40	0.66	7.80	2.60	41.3	131.2
	30–60	1.29	8.64	9.60	0.51	4.95	2.70	25.8	107.1
	60–80	1.30	6.21	11.30	0.50	3.40	2.10	16.0	118.5

### Experimental design

The experiment was arranged in a randomized block design with two factors (2 mulch levels × 6 N fertilizer application rates) and four replicates, and each plot measured 32 m^2^ (4 m × 8 m). Six N fertilizer application rates were assessed: 0 kg N ha^−1^ (N0), 80 kg N ha^−1^ (N80), 160 kg N ha^−1^ (N160), 240 kg N ha^−1^ (N240), 350 kg N ha^−1^ (N350) and 450 kg N ha^−1^ (N450). These N rates were selected from a wide range of local fertilizer application rates and considering prior research in this region^20^. Prior to mulching, each treatment received half of the total amount of N fertilizer (N80, 40 kg N ha^−1^; N160, 80 kg N ha^−1^; N240, 120 kg N ha^−1^; N350, 175 kg N ha^−1^; and N450, 225 kg N ha^−1^) as a basal fertilizer as well as 80 kg of P_2_O_5_ ha^−1^ and 80 kg of K_2_O ha^−1^, which were applied at the same time in both years. All of the plots were then prepared with 30-cm rows at a 60-cm line spacing. For the plastic mulch treatment, the entire ridge surface was immediately covered after preparation with colorless, transparent, 80-cm-wide and 0.008-mm-thick polyethylene film. After punching new holes in the ridges at distances of 40 cm, a maize seed (*Zea mays* L., cv. ‘Shandan 609’) was manually placed in each hole on April 20, 2015 (29 days after ridges were formed and mulched), and April 16, 2016 (20 days after ridges were formed and mulched). Weeds, diseases and insect pests were rare at the field site; thus, chemical control was not necessary. The purpose of preparing and mulching the ridges and furrows earlier than the maize sowing was to conserve soil moisture by reducing soil evaporation in the mulched plots. The experiment was implemented in the same field in 2015 and 2016, and each year, eight rows of maize were hand sown in each plot for a planting density of 60000 plants ha^−1^. This planting density is used by farmers in the region according to the recommendation of the local agricultural extension agency.

Each treatment received the other half of the total amount of N fertilizer (N80, 40 kg N ha^−1^; N160, 80 kg N ha^−1^; N240, 120 kg N ha^−1^; N350, 175 kg N ha^−1^; and N450, 225 kg N ha^−1^) as a top dressing with artificial fertilization, which was applied to all the treatments in early July. The maize crop was harvested on October 5, 2015, and October 12, 2016. After harvest, the plastic film was gathered and recycled by the manufacturer, and the soil was then plowed to a depth of 20 cm.

### Weather conditions and water use efficiency

**Figure 1:**
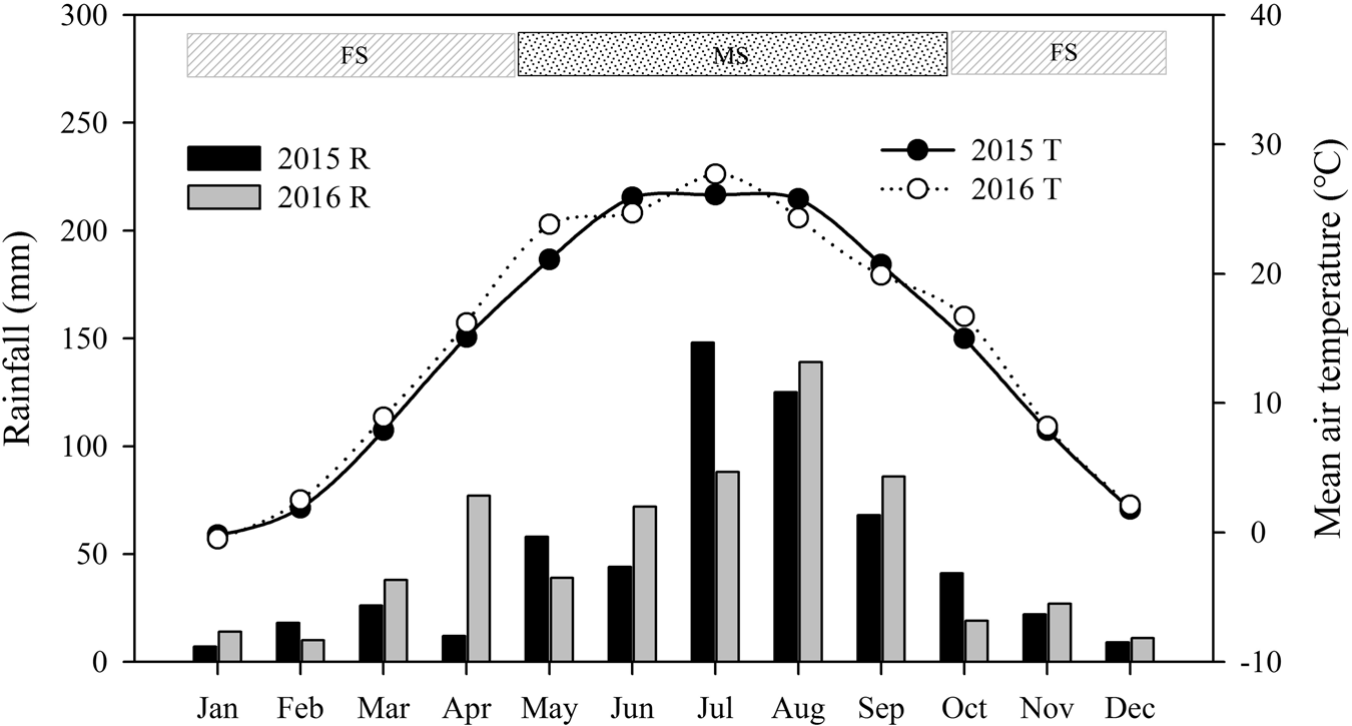
Distribution of monthly rainfall and mean air temperature at the experimental site in 2015 and 2016. MS and FS: the maize growing season and fallow season, respectively.

In each growing season, the amount of precipitation during each rainfall event at the site was measured using a rainfall recorder (wi92859, Dongxi Instrument Technology Ltd., Beijing, China) to calculate the total annual amount. The air temperature was recorded at the nearest weather station, which is located approximately 10 km from the experimental site and at 1069 m above sea level. The total precipitation was 578 mm and 620 mm in 2015 and 2016 (Fig. 1), respectively, but the distribution varied considerably between the two years. The winter and spring fallow season lasted approximately 170 days from the beginning of October to mid-April, and the highest precipitation occurred in July in 2015 and in August in 2016. The main cropping system in this area includes harvesting one crop of maize or potato per year. The amounts of rainfall during the fallow season were 123 mm and 119 mm in 2015 and 2016, respectively, and the amounts of rainfall during the maize growing season were 455 mm and 501 mm.

At 12 days before sowing and harvesting (168 days after seeding (DAS)) in 2015 and at 9 days before sowing and harvesting (179 DAS) in 2016, the soil water contents were measured every 20 cm to a depth of 200 cm. Four replicate soil samples were taken from each plot using an auger (25-mm inner diameter), and the soil water content in each soil layer was determined by weighing and drying the soil sample to a constant weight at 105 °C. The soil water contents were then converted to soil water storage (mm) using the soil bulk density. The soil water storage in the soil profile was the sum of the ten soil layers.

The total evapotranspiration (*ET*) during the entire maize growing season was calculated using the following equation^34^:

1 $$ET=P+I+{\rm{\Delta }}SW-S-D$$

where *P* is precipitation (mm); *I* is the amount of irrigation, which was equal to 0 because the experiment was performed with no irrigation treatment; *∆SW* is the change in soil water storage (mm) before sowing and after harvest; *S* is the upward flow into the root zone, which was negligible because the groundwater table was located at a depth of 80 m below the surface of the earth; and *D* is the deep seepage, which was negligible.

Water use efficiency (*WUE*, kg m^−3^) is equal to GY divided by the total *ET*^35^.The rainfall use efficiency (*RUE*, kg m^−3^) is equal to GY divided by the total rainfall during the maize growing season and fallow season^14^.

### Plant sampling and analysis

Four replicate plant samples were harvested close to the ground in each plot at the jointing stage (JS, 7-leaf stage), tasseling stage (TS, 14-leaf stage), silking stage (SS, silks visible outside the husks) and physiological maturity stage (PMS, at 42 days and 45 days after pollination in 2015 and 2016, respectively). The sampled plants were separated into stems (including sheaths and stems), leaves, bracts, ear axes and grains, and all the fallen leaves were carefully collected. During the harvest session, two rows in the middle of each plot were manually harvested to determine the GY. The plant samples were collected nearly one week later from the nonmulched treatments than from the mulched treatments because the plastic mulch advanced the maize development. The roots remaining in the soil were plowed *in situ* and mixed into the soil after harvest in both years.

After each sample collection period, all of the fresh plant organs were first heat-treated at 105 °C for 30 min, oven-dried at 75 °C to a constant weight, weighed to obtain the dry matter weight and milled into a fine powder for N measurements. The total N concentration in each organ was analyzed using the Kjeldahl method^36^.

### Calculations

The different N uptake and NT parameters were calculated as follows^36–39^:

2 $$PNU=\frac{DMA\times NC}{1000}$$

where *PNU* is the plant total N uptake (kg ha^−1^), *DMA* is the plant dry matter accumulation (kg ha^−1^), and *NC* is the N concentration (mg g^−1^).

3 $$TNT=TNA \mbox{-} SS-TNA \mbox{-} {\rm{PMS}}$$

where *TNT* is the total NT (kg ha^−1^), *TNA-*SS is the total N accumulation at the SS (kg ha^−1^), and *TNA-*PMS is the sum of the total N accumulation in the stems, sheaths, leaves, bracts and ear axis at the PMS (kg ha^−1^).

4 $$TNTE=\frac{Total\,nitrogen\,translocation}{Total\,nitrogen\,accumulation\,at\,SS}\times 100$$

where *TNTE* is the total NT efficiency (NTE) (%).

5 $$NAAS=PNU \mbox{-} {\rm{PMS}}-PNU \mbox{-} {\rm{SS}}-TNT$$

where *NAAS* is the N assimilation amount after the SS (kg ha^−1^), *PNU-*PMS is the plant total N uptake at the PMS (kg ha^−1^), and *PNU-*SS is the plant total N uptake at the SS (kg ha^−1^).

6 $$NHI=\frac{Total\,N\,in\,grain}{Total\,N\,in\,aboveground}\times 100$$

where *NHI* is the N harvest index (%).

7 $$NRE=\frac{PNU \mbox{-} {{\rm{PMS}}}_{{\rm{F}}}-PNU \mbox{-} {{\rm{PMS}}}_{{\rm{Z}}}}{NAP}\times 100$$

where *NRE* is the N apparent recovery efficiency (%), *PNU-*PMS_F_ is the plant total N uptake in the fertilization plot at the PMS (kg ha^−1^), and *PNU-*PMS_Z_ is the plant total N uptake in the nonfertilization plot at the PMS (kg ha^−1^).

*PFP* is the partial factor productivity of the fertilizer (kg kg^−1^), which is equal to GY divided by the sum of N, phosphate and potassic fertilizer applications during each crop growing season (kg ha^−1^).

### Statistical analyses

Three-way ANOVA with the N application rate, plastic mulch and cropping year as three fixed factors was performed to assess variations in the maize PNU at the JS (PNU-JS), PNU at the TS (PNU-TS), PNU at the SS (PNU-SS), PNU at the PMS (PNU-PMS), NT, NTE, NAAS, NHI, NUE, NRE and PFP (Supplementary Tables 1–14) One-way ANOVA was conducted to evaluate aboveground N accumulation of maize crops at the JS, TS, SS, and PMS and N accumulation and NTE in different organs. The differences between all treatments were detected using least significant difference (LSD) tests at the 0.05 significance level. Statistical analyses and data plotting were performed using SPSS Statistics Software 16.0 and Sigma Plot 10.0, respectively.

## Results

### Grain yield, water use efficiency, and rainfall use efficiency

**Figure 2:**
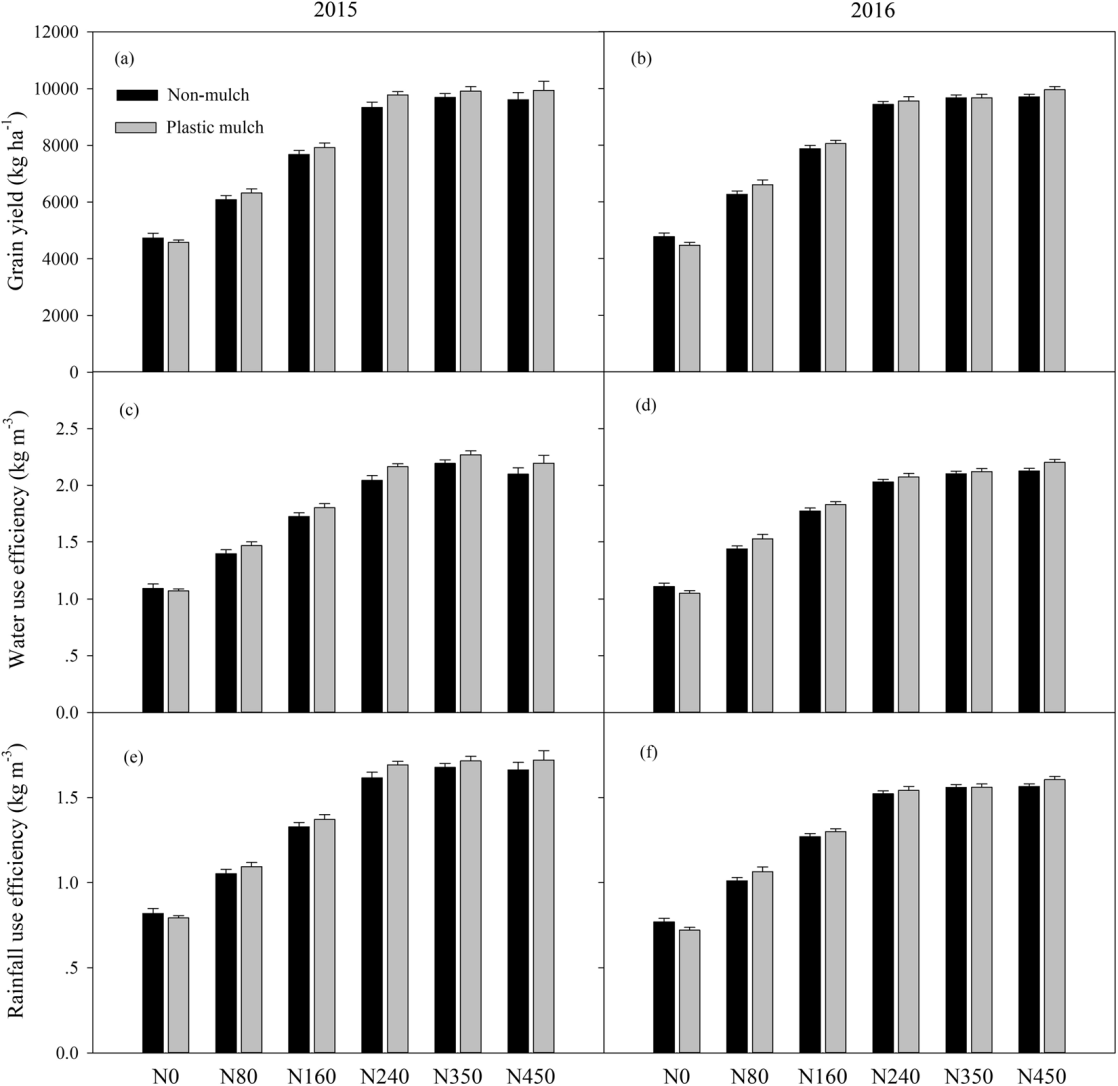
Variations in the grain yield (**a**,**b**), water use efficiency (**c**,**d**) and rainfall use efficiency (**e**,**f**) with changes in the N fertilizer level with and without mulching in 2015 and 2016. The bars are the mean + one standard error of the mean (n = 4).

There were significant interactions between year and N fertilizer rate and between N fertilizer rate and mulch on the maize GY, and both N fertilizer rate and mulch had a significant effect on the GY (Fig. 2a,b; Table 2). In 2015, the GY with the six N fertilizer rates varied from 4733 to 9693 kg ha^−1^ without mulch and from 4582 to 9942 kg ha^−1^ with mulch. In 2016, the GY varied from 4773 to 9700 kg ha^−1^ without mulch and from 4470 to 9954 kg ha^−1^ with mulch. Under mulch treatments, the highest GY was observed with the N450 treatment, and this value was 53.9%, 36.4%, 20.2%, 1.6% and 0.3% higher than those obtained with the N0, N80, N160, N240 and N350 treatments in 2015, respectively (Fig. 2a). Similar results were observed in 2016, but a variation was observed compared with the no-mulch treatments in 2015. In two consecutive years, mulch increased GY for the same N fertilizer applied, but the GY was higher in the no-mulch treatment (4733 kg ha^−1^) than in the mulch treatment (4582 kg ha^−1^) under N0 in 2015 (Fig. 2a). Similarly, the GY was higher in the no-mulch treatment (4772 kg ha^−1^) than in the mulch treatment (4470 kg ha^−1^) under N0 in 2016 (Fig. 2b).

**Table 2:** Treatment effects (*p* values) on maize plant total nitrogen uptake at the jointing stage (PNU-JS), tasseling stage (PNU-TS), silking stage (PNU-SS) and physiological maturity stage (PNU-PMS), nitrogen translocation (NT), nitrogen translocation efficiency (NTE), grain yield (GY), water use efficiency (WUE), rainfall use efficiency (RUE), nitrogen assimilating amount after SS stage (NAAS), nitrogen harvest index (NHI), nitrogen use efficiency (NUE), nitrogen apparent recovery efficiency (NRE) and partial factor productivity of the fertilizer (PFP), using cropping year (Y), nitrogen fertilizer levels (N) and plastic mulch (M) as three fixed factors.

Source	PNU-JS	PNU-TS	PNU-SS	PNU-PMS	NT	NTE	GY	WUE	RUE	NAAS	NHI	NUE	NRE	PFP
Year (Y)	<0.001	<0.001	<0.001	<0.001	0.048	0.001	ns	ns	<0.001	0.003	ns	0.003	ns	0.018
N fertilizer (N)	<0.001	<0.001	<0.001	<0.001	<0.001	<0.001	<0.001	<0.001	<0.001	<0.001	<0.001	<0.001	<0.001	<0.001
Mulch (M)	<0.001	<0.001	<0.001	<0.001	<0.001	<0.001	<0.001	<0.001	<0.001	ns	<0.001	<0.001	ns	<0.001
Y × N	<0.001	<0.001	<0.001	<0.001	0.008	<0.001	0.009	<0.001	<0.001	<0.001	<0.001	<0.001	0.002	<0.001
Y × M	0.04	0.001	ns	ns	0.034	0.017	ns	0.024	0.036	ns	0.001	ns	ns	ns
N × M	ns	<0.001	ns	ns	ns	<0.001	<0.001	<0.001	<0.001	<0.001	<0.001	<0.001	ns	0.029
Y × N × M	ns	<0.001	ns	ns	ns	<0.001	ns	ns	ns	0.003	0.015	ns	ns	ns

ns: not significant (*p > *0.05).

The interactions between any two of the factors of year, mulch and N fertilizer rate had significant effects on the WUE and RUE (Fig. 2c,d; Table 2). The WUE increased with increases in the N fertilizer rates and with increases in the GY, but the speed increased first and then decreased with the increased N fertilizer rate. The highest WUE was obtained in the N350 treatment without mulching, and this value was 51.3%, 37.3%, 20.9% and 3.8% higher than those obtained with the N0, N80, N160 and N240 treatments in 2015, respectively. The N450 rate did not continue to improve the WUE in 2015. Plastic mulching increased the WUE, and the average WUEs under plastic mulching were 4% and 2.1% higher than those without mulching throughout all the treatments in 2015 and 2016, respectively. The RUE rates with different N fertilizer application rates were ranked as N350 > N450 > N240 > N160 > N80 > N0 in 2015, and the average RUE under the N350 treatment was 0.89 kg m^−3^, 0.62 kg m^−3^, 0.35 kg m^−3^, 0.04 kg m^−3^ and 0.01 kg m^−3^ higher than those under the N0, N80, N160, N240 and N450 treatments, respectively (Fig. 2e,f). However, the N450 treatment had a stronger effect than the other treatments in 2016.

### Crop organ nitrogen accumulation, translocation and translocation efficiency and nitrogen assimilation amount after the silking stage

**Figure 3:**
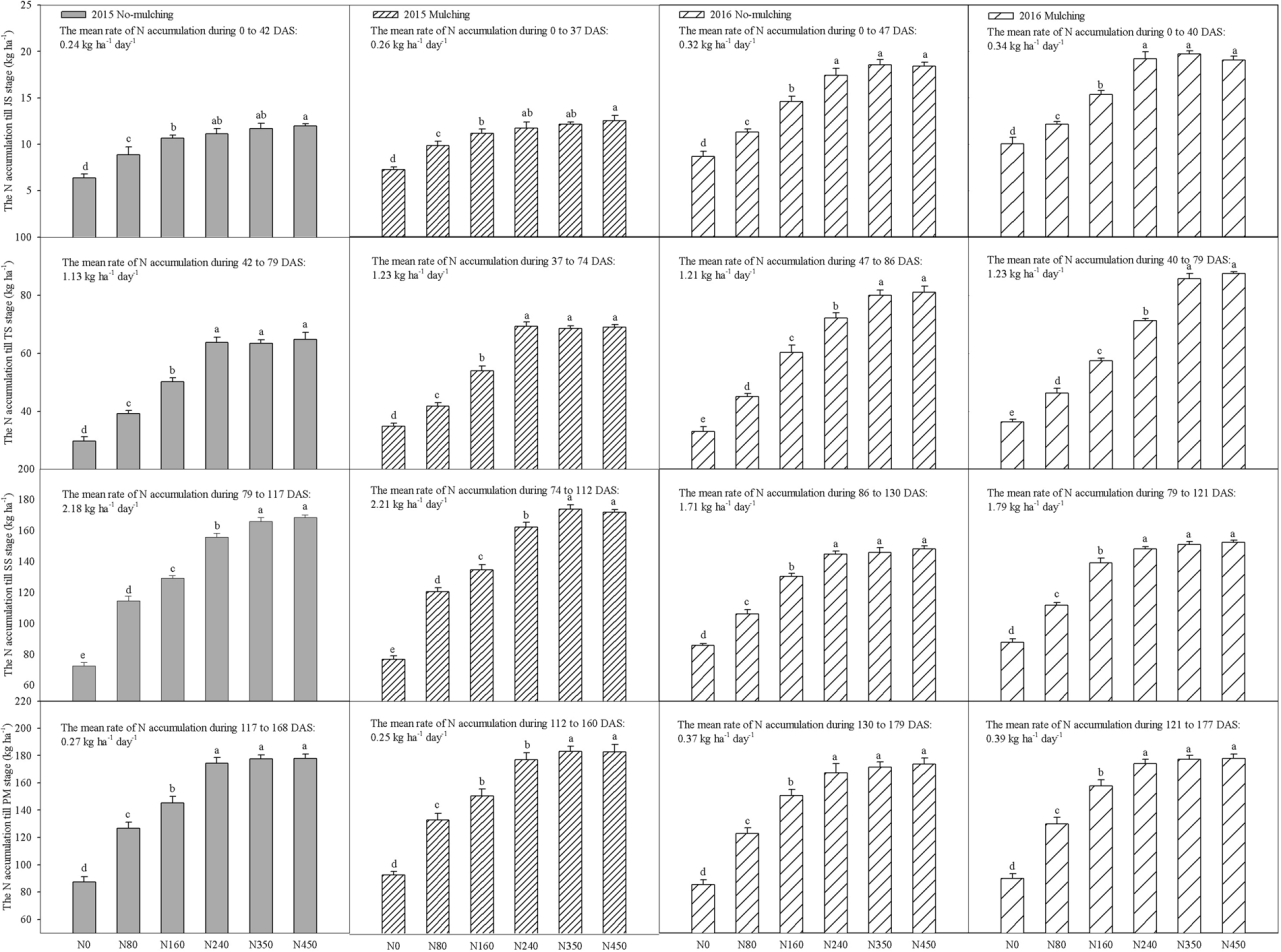
Aboveground N uptake by maize crops based on the N fertilizer rate at different growth stages in 2015 and 2016. JS, jointing stage; TS, tasseling stage; SS, silking stage; PMS, physiological maturity stage. The bars are the mean + one standard error of the mean (n = 4). Different letters indicate significant differences among the N fertilizer rates (p < 0.05).

Over the entire growing season, the PNU significantly increased (*p* < 0.05) with increases in the N application rates with and without plastic mulch in the two consecutive years (Fig. 3). The individual factors of year and mulch significantly affected the PNU at different growth stages (Table 2), but there was no significant difference among N240, N350 and N450 during most of the growth stages in both years (Fig. 3). The mean PNU-JS values obtained with the plastic mulch treatment were 6.5% and 7.5% higher than those obtained with the no-mulch treatment in 2015 and 2016, respectively, and similar results were also observed in the TS, SS and PMS. With plastic mulching, the two-year average PNU values up to the PMS with the N450 treatment were 0.1%, 2.7%, 14.5%, 27.1% and 49.4% higher than those with the N350, N240, N160, N80 and N0 treatments, respectively. In particular, with plastic mulching, the value obtained with N350 was slightly higher than that obtained with N450 at the PMS in 2015. Similarly, in the absence of mulching, the values obtained with N450 were 0.8%, 2.9%, 15.8%, 29% and 50.8% higher than those obtained with N350, N240, N160, N80 and N0, respectively.

**Figure 4:**
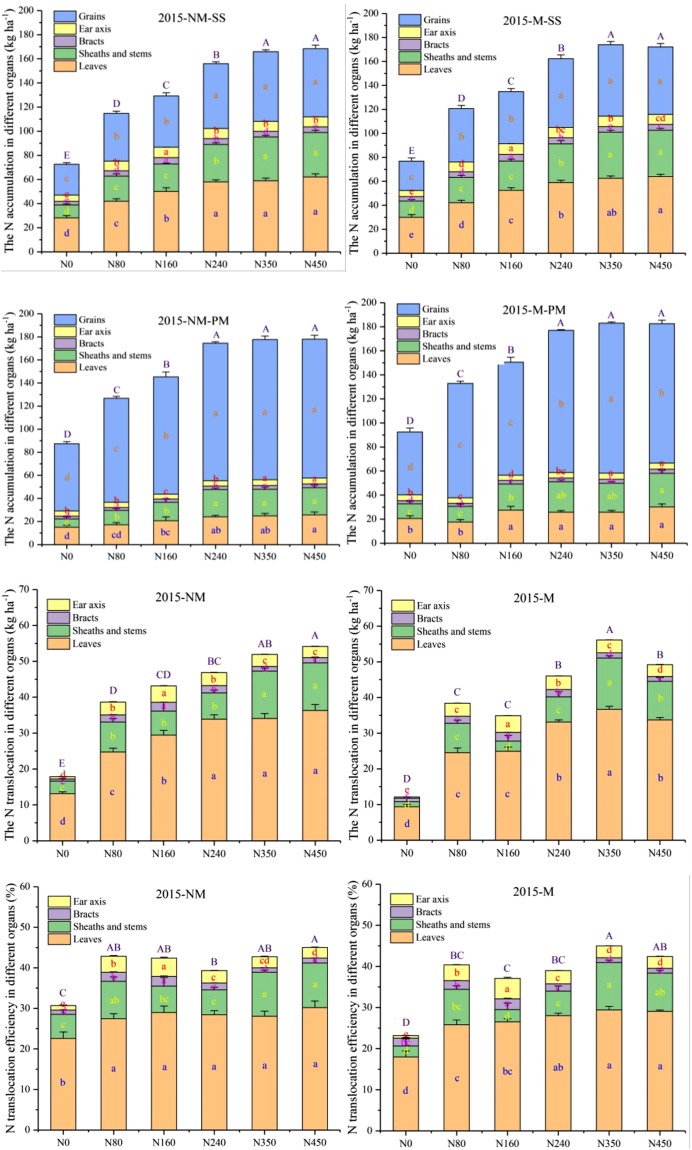
The N accumulation, N translocation and N translocation efficiency in different organs at the silking and physiological maturity stages were affected by plastic mulching and the N fertilizer application rate in 2015. NM: no-mulch treatment; M: plastic mulch treatment; SS: silking stage; PMS: physiological maturity stage. The bars are the mean + one standard error of the mean (n = 4). Different lowercase letters indicate significant differences among the N fertilizer rates (p < 0.05), and capital letters indicate significant differences among the effects of the N fertilizer rates on total N accumulation, N translocation and N translocation efficiency (p < 0.01).

**Figure 5:**
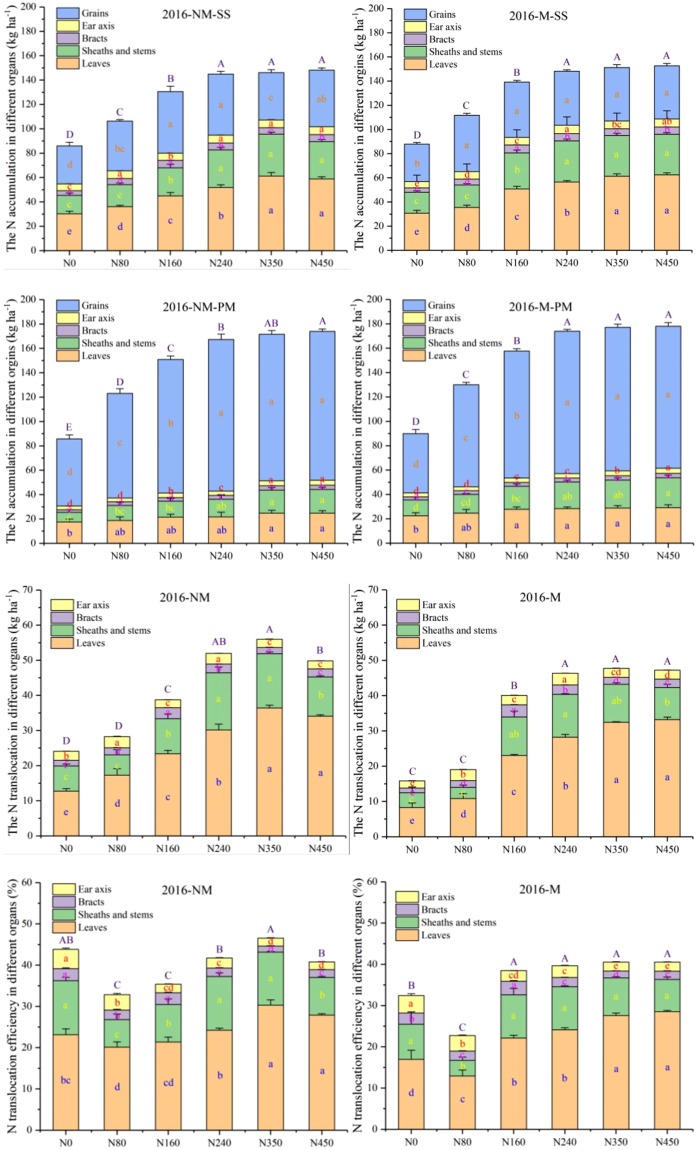
The N accumulation, N translocation and N translocation efficiency in different organs at the silking and physiological maturity stages were affected by plastic mulching and the N fertilizer application rate in 2016. NM: no-mulch treatment; M: plastic mulch treatment; SS: silking stage; PMS: physiological maturity stage. The bars are the mean + one standard error of the mean (n = 4). The different lowercase letters indicate significant differences among the N fertilizer rates (p < 0.05), and capital letters indicate significant differences among the effects of the N fertilizer rate on the total N accumulation, N translocation and N translocation efficiency (p < 0.01).

The crop organ N accumulation, NT and NTE at the SS and PMS in different organs were affected by plastic mulching and N fertilizer application rate in 2015 and 2016 (Figs 4, 5). The total crop organ N accumulation values obtained with the N450 and N350 treatments were significantly (*p* < 0.01) higher than those obtained with the other treatments at the SS, and there was no significant difference among N240, N350 or N450 treatments at the PMS in 2015 (Fig. 4). Across the treatments, the average N accumulation in the leaves with plastic mulching was 13.5% and 20.1% higher than that obtained with the no-mulch treatment at the PMS in 2015 and 2016, respectively. Similar results were observed in the grains, sheaths and stems, but the N accumulation in the principles of the bracts and ear axis was clearly different. Across treatments, the average leaf N accumulation in the leaves with plastic mulching was 13.5% and 20.1% higher than that obtained with the no-mulch treatment at the PMS in 2015 and 2016, respectively. Interestingly, the average leaf N translocation (LNT) with plastic mulching was 5.7% and 13.3% lower than that obtained with the no-mulch treatment from the SS to the PMS in 2015 and 2016, respectively.

The N fertilizer treatments significantly increased the total amount of NT as well as the N accumulation in both years (*p* < 0.01) (Figs 4, 5). There was a significant three-way interaction effect among year, N fertilizer rate and mulch and a two-way interaction effect between N fertilizer rate and mulch on NTE (Figs 4, 5; Table 2). Over the two consecutive years, the average NT in different organs generally followed the order of leaves (26 kg ha^−1^) >sheaths and stems (8.9 kg ha^−1^) >ear axis (2.9 kg ha^−1^) >bracts (2 kg ha^−1^). The effect of plastic mulching on the total NTE was similar to that on NT in 2015 (Fig. 4), but the total NTE with the N0 treatment was significantly (*p* < 0.01) higher than that with the N80 treatment with and without plastic mulch in 2016 (Fig. 5). However, the LNTE was increased with increases in the N fertilizer rates in the two consecutive years. Averaging across the N fertilizer rates, the NTE under the no-mulch treatment was 5.6% and 12.9% higher than that under the plastic mulch treatment from the SS to PMS in 2015 and 2016, respectively.

**Figure 6:**
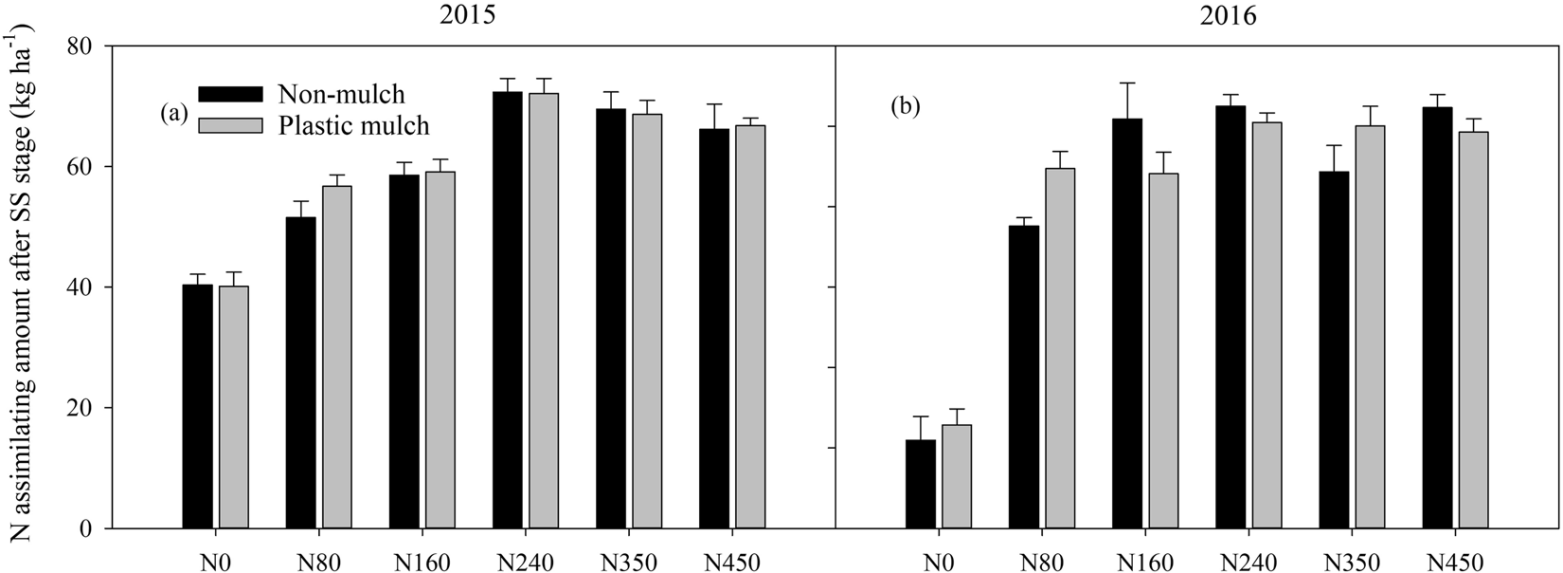
Variations in N assimilation amount after the silking stage (**a**,**b**) with changes in the N fertilizer level with and without mulching in 2015 and 2016. The bars are the mean + one standard error of the mean (n = 4).

On the NAAS, there was a significant three-way interaction among year, N fertilizer rate and mulch and two-way interactions between year and mulch and between N fertilizer rate and mulch (Fig. 6a,b; Table 2). Specifically, mulching did not significantly affect the N assimilation amount (Table 2). In 2015, the highest NAAS values were obtained with the N240 treatment, and the average increases in N assimilation amount were 44.2%, 28.8%, 3.9% and 8.5% compared with those obtained with the N0, N80, N160, N350 and N450 treatments without mulching, respectively (Fig. 6a). Similar results were also obtained in 2016 (Fig. 6b). The N assimilation amount under the plastic mulch treatments was 11.8% and 7.9% higher than that under the no-mulch treatments in 2015 and 2016, respectively (Fig. 6a,b).

### Nitrogen harvest index, nitrogen use efficiency, nitrogen apparent recovery efficiency and partial factor productivity of the fertilizer

**Figure 7:**
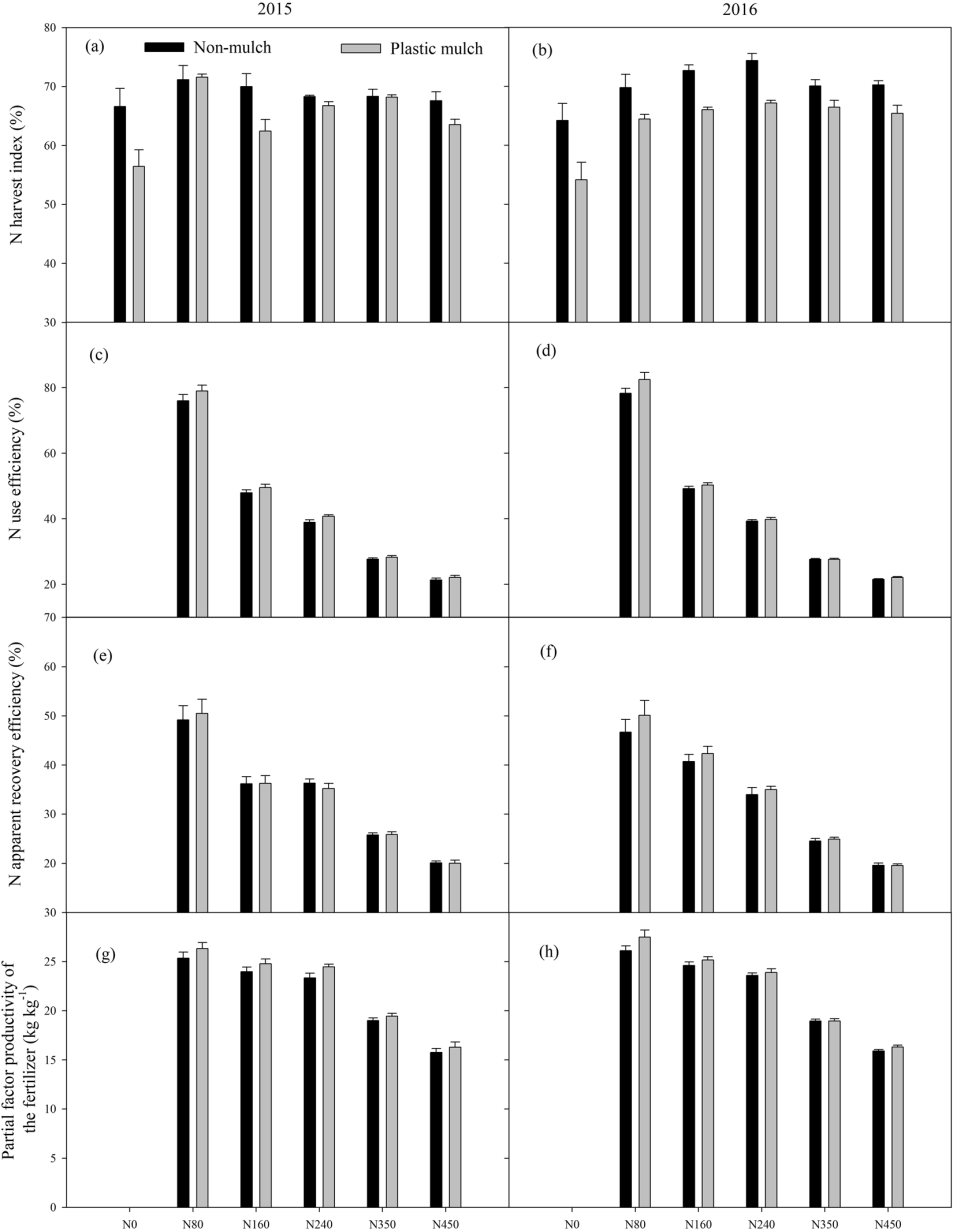
Variations in the N harvest index (**a**,**b**), N use efficiency (**c**,**d**), N apparent recovery efficiency (**e**,**f**) and partial factor productivity of the fertilizer (**g**,**h**) with changes in the N fertilizer rates with and without mulching in 2015 and 2016. The bars are the means + one standard error of the mean (n = 4).

Analysis of the NHI revealed a significant three-way interaction effect among year, N fertilizer rate and mulch and two-way interactions between year and mulch and between N fertilizer rate and mulch, but year had no significant effect on NHI (Table 2). Plastic mulch reduced the NHI in both years, and averaged across the N fertilizer rates, the NHI with plastic mulching was 5.9% and 9.8% lower than that without mulch in 2015 and 2016, respectively (Fig. 7a,b).

There was a significant interaction effect among year, N fertilizer rate and mulch and two-way interaction effects between year and mulch and between N fertilizer rate and mulch on the NUE (Fig. 7c,d; Table 2). The N fertilizer application rate decreased the NUE both years, but mulching increased the NUE (Fig. 7c,d). The NUE with different N fertilizer rates followed the order of N80 > N160 > N240 > N350 > N450, and N80 increased the average NUE by 28.1%, 37.1%, 48.4% and 54.7%, respectively, without mulching in 2015 (Fig. 7c). Averaging across the N fertilizer rates, mulching increased the NUE in both years, and the NUE values with plastic mulch treatment were 3.5% and 2.9% higher than those obtained without mulching in 2015 and 2016, respectively (Fig. 7c,d).

The interaction between year and N fertilizer rate and the N fertilizer rate alone had significant effects on the NRE (*p* < 0.05, Fig. 7e,f; Table 2). Thus, the N80 treatment yielded the highest NRE without and with mulching in both years, whereas N450 consistently showed the lowest NRE. However, the NRE obtained with plastic mulching was only 1.8% and 3.9% higher in 2015 and 2016 than that obtained without mulching, respectively (Fig. 7e,f). Mulch generally increased the PFP in the two consecutive years (Fig. 7g,h). Averaging across years, the N80 treatment with and without mulching resulted in a greater PFP than the other treatments, but the differences in the PFP between the mulch and no-mulch treatments were not significant in 2015 and 2016. Only the N fertilizer rate had a significant effect on the PFP in both years (Table 2).

## Discussion

### Nitrogen uptake, translocation and translocation efficiency

Our data show that N fertilizer application significantly increased the uptake of N by plants in the two consecutive years, but no significant difference was obtained with N fertilizer rates greater than 240 kg ha^−1^. The application of N fertilizer to crops is essential for enhancing N uptake and GY, which is consistent with the results of most previous studies^40–42^. When the yield and soil residual N are considered together, the optimal N fertilizer under field conditions ranges from 100 to 240 kg ha^−1 ^^43^, which also supports the results of our research.

Plastic mulch increased the GY and plant N accumulation under all of the N fertilizer rates tested in this study. While the benefits of mulching have been well documented^44^, the present study showed that the effect of mulch on plant N accumulation, NT and NTE varied with the N fertilizer rate. In the nonmulched plots in 2015, N450 resulted in the highest plant N accumulation, whereas N0 had the lowest yield; in the mulched plots, the highest plant N accumulation was obtained with the N350 treatment. Some of the accumulated N in grain arises from new N assimilation during reproductive growth, but the rest must be supplemented by the NT from the plant vegetative organs^45^. The NT observed in this study was similar to the plant N accumulation, but mulch had no significant effect on the NT. The higher N accumulation in the TS will reduce NT from the leaf to the grain^46^, which could explain why the NTE was not consistent with the N fertilizer application rates.

Surprisingly, the NTE does not increase with increases in the N fertilizer rates. Moreover, mulch reduced the NTE in both years. Therefore, the NTE before reproductive growth appears to be regulated by both the environment and genotype^47–49^, which suggests that using plastic mulch increases the stability of plant N accumulation in the face of variable NTE across the N fertilizer rates. Our results showed that the interaction of year and the N fertilizer application rate had a significant effect on N uptake and NT. There may be two reasons for this: first, the distribution of rainfall in the maize growing season varied considerably between the two years for the same month (Fig. 1), and second, CO_2_ concentrations in the atmosphere could be another reason for the significant differences in N uptake and NT between the two years^50^.

Greater NT is observed when the plant N accumulation is less than the grain N requirements, and 33% and 22% of the grain N is contributed by the leaf and supplied from the stem at maturity, respectively^38^. In 2015, the grain N translocated from the leaf ranged from 27.5% to 30.2% under the N fertilizer treatments in the plots without mulching and from 25.8% to 29.4% in the mulched plots. In 2016, much rainfall was concentrated in the early stages, which induced higher N accumulation at SS in all treatments, with or without mulch, compared with that obtained in 2015, and the increases in the LNTE without mulch relative to with mulch across the N fertilizer rates were greater in 2015 than in 2016. The lower LNTE obtained in this study might have been caused by the different calculation time interval of the leaf NT, year, location and genotype^51^.

The ranking of NT for grain N accumulation was clearly leaves > sheaths and stems > ear axis > bracts, which is consistent with the results reported by Wang *et al*.^36^. This is similar to the conclusion that a larger amount of the N accumulated in the grain was translocated from the leaf and stem during the grain-filling stage, and slightly more of the leaf N was translocated into the grain than into the stem^37^. This is potentially because the leaf is the main organ for maize photosynthesis, resulting in a higher N concentration before PMS. Therefore, after considering the NTE across cropping years and mulch treatments, N240 was considered the most suitable N fertilizer rate in the experiments to increase grain N accumulation both with and without plastic mulch.

### Grain yield, water use efficiency and nitrogen use efficiency

Many studies have proven that mulch and N fertilizer application lead to higher maize yields^52^. The yield reached a relatively high value when the N fertilizer rate was greater than 240 kg ha^−1^ with and without plastic mulch, and there was no significant difference among the N240, N350 and N450 treatments. The yield decreases with reductions in the amount of N fertilizer applied^53^. However, the mulch was not able to increase the GY without fertilizer, which may indicate that there is an important synergistic relationship between mulch and N fertilizer application. Therefore, the necessary and sufficient condition for plastic mulch to increase the GY is adequate soil nutrients. The WUE and RUE had slightly beneficial effects on the GY under mulch treatments, and the yields with plastic mulch were higher because of the higher solar radiation and soil temperature during early growth stages and the slightly higher WUE of soil under mulch than in that without mulch^54–56^. The relatively lower water consumption and higher GY obtained with the N350 treatment resulted in the highest WUE and RUE. Therefore, considering the water savings alone, N350 was the best N fertilizer rate for maintaining yield.

A lower amount of N fertilizer enhanced the N assimilation amount and NHI under the no-mulch treatments, which might be related to the physiological mechanisms limiting plant N uptake capacity^57^. The higher N fertilizer rate corresponded to higher N accumulation before SS, and the lower NAAS was as expected. In addition, within an appropriate range of N application rates, increased application of N fertilizer yielded higher NUE, NRE and PFP values with the plastic mulch and no-mulch treatments (Fig. 7). Normally, higher NUE, NRE and PFP values were obtained with mulch than without mulch, and this difference might be explained by an increase in the soil heat available to maize crops, which is important for generating yield in this region^58^. However, whether plastic mulch improves the NUE might also depend on its function in enhancing soil organic matter and modifying soil environmental conditions^59^. In our study, the NUE ranged from 21.4% to 76.1% with N application rates from 80 kg ha^−1^ to 450 kg ha^−1^, and these results agree with the findings of previous studies that showed a maximum NUE of 78% with different amounts of applied N under N deficiency^60^.

We hypothesized that plastic mulch combined with N fertilizer application would play a very important role in maize yield as well as the PNU, WUE, NUE, and NHI, among other factors. However, the results showed that the mulch effectively increased the PNU before SS, particularly under the premise of an N fertilizer application rate less than 240 kg ha^−1^. The NHI was markedly different from what was expected. The N fertilizer rate at 350 kg ha^−1^ both with plastic mulch and without came closest to reaching the highest GY in the two consecutive years in this study. Nevertheless, this result considered the relationship between the N fertilizer rate and GY with and without plastic mulch. In practice, more N fertilizer is generally used by small farmers than by commercial producers. Therefore, high doses of N fertilizers are used for vegetable crops in this region. Reducing the N application rates and optimizing the fertilization time as much as possible can reduce the risk of yield reduction, which could itself reduce the negative environmental impacts of N application^61,62^.

## Conclusion

Plastic mulching increased the GY and plant total N uptake at the JS, TS, SS and PMS and the NT, NTE, NUE, partial factor productivity of the fertilizer, WUE, and RUE of the grain. However, the N assimilation amount after the silking stage and the N harvest index decreased with increases in the N fertilizer rates. In particular, the effects of plastic mulching on the GY and the various indicators varied significantly with changes in the N fertilizer rate. We conclude that an N fertilizer application rate of 240 kg ha^−1^ with mulch can achieve relatively higher NTE, GY, WUE and NUE. Whether a large N fertilizer rate favored an optimal application was not clear because the variations in the soil environment and economic benefits were not entirely consistent with those of the combined evaluation in this study. Additionally, N assimilation and the N harvest index were negatively related to the N fertilizer rate; thus, N assimilation and gain by the plants were not associated with plastic mulching among the different N fertilizer rates.

## Electronic supplementary material

Supplementary Information
