# Supplementary material for: Synergetic effects of plastic mulching and nitrogen application rates on grain yield, nitrogen uptake and translocation of maize planted in the Loess Plateau of China
Source: Sci Rep. 2018 Sep 25;8:14319. doi: 10.1038/s41598-018-32749-9 (PMC6156601; doi:10.1038/s41598-018-32749-9)
Supplement: Supplementary file 1 — Supplementary Information [file 41598_2018_32749_MOESM1_ESM.doc]

**Supplementary Information (SI)**

**Synergetic effects of plastic mulching and nitrogen application rates on grain yield, nitrogen uptake and translocation of maize planted in the Loess Plateau of China**

Xiukang Wang^1^, Ning Wang^1^, Yingying Xing^1^, Mohamed Ben El Caid^2^

**Author affiliations:**

^1^College of Life Sciences, Yan'an University, Yan'an, Shaanxi 716000, China

^2^Laboratory of Biotechnology and Valorization of Natural Resources, Department of Biology, Faculty of Science, Ibn Zohr University, 8106, Agadir, Morocco

**Corresponding author:**

College of Life Sciences, Yan'an University, Yan'an, Shaanxi 716000, China

wangxiukang@126.com (Xiukang Wang)

Telephone and fax: +86 9112332020

Supplematary material and methods

Supplement Tables: 14

Supplementary Table S1. Analysis of variance summary for maize plant total nitrogen uptake at jointing stage (PNU-JS) as affected by cropping year (Y), nitrogen fertilizer levels (N) and plastic mulch (M) as three fixed factors.

| **Tests of Between-Subjects Effects** | | | | | |
| --- | --- | --- | --- | --- | --- |
| Dependent Variable: PNU-JS | |  |  |  |  |
| Source | Type III Sum of Squares | df | Mean Square | F | Sig. |
| Corrected Model | 1439.740^a^ | 23 | 62.597 | 233.222 | .000 |
| Intercept | 16006.335 | 1 | 16006.335 | 5.964E4 | .000 |
| Y | 580.167 | 1 | 580.167 | 2.162E3 | .000 |
| M | 18.904 | 1 | 18.904 | 70.431 | .000 |
| N | 738.294 | 5 | 147.659 | 550.139 | .000 |
| Y * M | 1.170 | 1 | 1.170 | 4.361 | .040 |
| Y * N | 98.855 | 5 | 19.771 | 73.661 | .000 |
| M * N | 1.257 | 5 | .251 | .937 | .462 |
| Y * M * N | 1.093 | 5 | .219 | .815 | .543 |
| Error | 19.325 | 72 | .268 |  |  |
| Total | 17465.400 | 96 |  |  |  |
| Corrected Total | 1459.065 | 95 |  |  |  |
| a. R Squared = .987 (Adjusted R Squared = .983) | | | |  |  |

Supplementary Table S2. Analysis of variance summary for maize plant total nitrogen uptake at tasseling stage (PNU-TS) as affected by cropping year (Y), nitrogen fertilizer levels (N) and plastic mulch (M) as three fixed factors.

| **Tests of Between-Subjects Effects** | | | | | |
| --- | --- | --- | --- | --- | --- |
| Dependent Variable: PNU-TS | |  |  |  |  |
| Source | Type III Sum of Squares | df | Mean Square | F | Sig. |
| Corrected Model | 27641.798^a^ | 23 | 1201.817 | 526.039 | .000 |
| Intercept | 328348.827 | 1 | 328348.827 | 1.437E5 | .000 |
| Y | 1935.010 | 1 | 1935.010 | 846.960 | .000 |
| M | 251.554 | 1 | 251.554 | 110.106 | .000 |
| N | 24450.688 | 5 | 4890.138 | 2.140E3 | .000 |
| Y * M | 26.882 | 1 | 26.882 | 11.766 | .001 |
| Y * N | 830.990 | 5 | 166.198 | 72.745 | .000 |
| M * N | 79.944 | 5 | 15.989 | 6.998 | .000 |
| Y * M * N | 66.731 | 5 | 13.346 | 5.842 | .000 |
| Error | 164.495 | 72 | 2.285 |  |  |
| Total | 356155.120 | 96 |  |  |  |
| Corrected Total | 27806.293 | 95 |  |  |  |
| a. R Squared = .994 (Adjusted R Squared = .992) | | | |  |  |

Supplementary Table S3. Analysis of variance summary for maize plant total nitrogen uptake at silking stage (PNU-SS) as affected by cropping year (Y), nitrogen fertilizer levels (N) and plastic mulch (M) as three fixed factors.

| **Tests of Between-Subjects Effects** | | | | | |
| --- | --- | --- | --- | --- | --- |
| Dependent Variable: PNU-SS | |  |  |  |  |
| Source | Type III Sum of Squares | df | Mean Square | F | Sig. |
| Corrected Model | 84741.467^a^ | 23 | 3684.412 | 876.625 | .000 |
| Intercept | 1707920.230 | 1 | 1707920.230 | 4.064E5 | .000 |
| Y | 1478.155 | 1 | 1478.155 | 351.695 | .000 |
| M | 666.233 | 1 | 666.233 | 158.516 | .000 |
| N | 79049.707 | 5 | 15809.941 | 3.762E3 | .000 |
| Y * M | 2.836 | 1 | 2.836 | .675 | .414 |
| Y * N | 3466.837 | 5 | 693.367 | 164.972 | .000 |
| M * N | 46.496 | 5 | 9.299 | 2.213 | .062 |
| Y * M * N | 31.203 | 5 | 6.241 | 1.485 | .205 |
| Error | 302.612 | 72 | 4.203 |  |  |
| Total | 1792964.310 | 96 |  |  |  |
| Corrected Total | 85044.080 | 95 |  |  |  |
| a. R Squared = .996 (Adjusted R Squared = .995) | | | |  |  |

Supplementary Table S4. Analysis of variance summary for maize plant total nitrogen uptake at physiological maturity stage (PNU-PM) as affected by cropping year (Y), nitrogen fertilizer levels (N) and plastic mulch (M) as three fixed factors.

| **Tests of Between-Subjects Effects** | | | | | |
| --- | --- | --- | --- | --- | --- |
| Dependent Variable: PNU-PM | |  |  |  |  |
| Source | Type III Sum of Squares | df | Mean Square | F | Sig. |
| Corrected Model | 101930.938^a^ | 23 | 4431.780 | 985.814 | .000 |
| Intercept | 2144786.882 | 1 | 2144786.882 | 4.771E5 | .000 |
| Y | 139.202 | 1 | 139.202 | 30.964 | .000 |
| M | 665.707 | 1 | 665.707 | 148.081 | .000 |
| N | 100682.408 | 5 | 20136.482 | 4.479E3 | .000 |
| Y * M | 5.415 | 1 | 5.415 | 1.205 | .276 |
| Y * N | 405.431 | 5 | 81.086 | 18.037 | .000 |
| M * N | 15.903 | 5 | 3.181 | .708 | .620 |
| Y * M * N | 16.872 | 5 | 3.374 | .751 | .588 |
| Error | 323.680 | 72 | 4.496 |  |  |
| Total | 2247041.500 | 96 |  |  |  |
| Corrected Total | 102254.618 | 95 |  |  |  |
| a. R Squared = .997 (Adjusted R Squared = .996) | | | |  |  |

Supplementary Table S5. Analysis of variance summary for nitrogen translation (NT) as affected by cropping year (Y), nitrogen fertilizer levels (N) and plastic mulch (M) as three fixed factors.

| **Tests of Between-Subjects Effects** | | | | | |
| --- | --- | --- | --- | --- | --- |
| Dependent Variable: NT | |  |  |  |  |
| Source | Type III Sum of Squares | df | Mean Square | F | Sig. |
| Corrected Model | 10005.473^a^ | 23 | 435.021 | 20.242 | .000 |
| Intercept | 228382.109 | 1 | 228382.109 | 1.063E4 | .000 |
| Y | 86.678 | 1 | 86.678 | 4.033 | .048 |
| M | 1789.517 | 1 | 1789.517 | 83.267 | .000 |
| N | 7360.208 | 5 | 1472.042 | 68.495 | .000 |
| Y * M | 100.696 | 1 | 100.696 | 4.685 | .034 |
| Y * N | 364.756 | 5 | 72.951 | 3.394 | .008 |
| M * N | 186.713 | 5 | 37.343 | 1.738 | .137 |
| Y * M * N | 116.905 | 5 | 23.381 | 1.088 | .374 |
| Error | 1547.366 | 72 | 21.491 |  |  |
| Total | 239934.948 | 96 |  |  |  |
| Corrected Total | 11552.839 | 95 |  |  |  |
| a. R Squared = .866 (Adjusted R Squared = .823) | | | |  |  |

Supplementary Table S6. Analysis of variance summary for nitrogen translation efficiency (NTE) as affected by cropping year (Y), nitrogen fertilizer levels (N) and plastic mulch (M) as three fixed factors.

| **Tests of Between-Subjects Effects** | | | | | |
| --- | --- | --- | --- | --- | --- |
| Dependent Variable: NTE | |  |  |  |  |
| Source | Type III Sum of Squares | df | Mean Square | F | Sig. |
| Corrected Model | 3604.622^a^ | 23 | 156.723 | 49.389 | .000 |
| Intercept | 142789.021 | 1 | 142789.021 | 4.500E4 | .000 |
| Y | 35.473 | 1 | 35.473 | 11.179 | .001 |
| M | 305.000 | 1 | 305.000 | 96.117 | .000 |
| N | 1487.286 | 5 | 297.457 | 93.740 | .000 |
| Y * M | 19.009 | 1 | 19.009 | 5.990 | .017 |
| Y * N | 1311.353 | 5 | 262.271 | 82.651 | .000 |
| M * N | 245.415 | 5 | 49.083 | 15.468 | .000 |
| Y * M * N | 201.085 | 5 | 40.217 | 12.674 | .000 |
| Error | 228.472 | 72 | 3.173 |  |  |
| Total | 146622.116 | 96 |  |  |  |
| Corrected Total | 3833.094 | 95 |  |  |  |
| a. R Squared = .940 (Adjusted R Squared = .921) | | | |  |  |

Supplementary Table S7. Analysis of variance summary for grain yield (GY) as affected by cropping year (Y), nitrogen fertilizer levels (N) and plastic mulch (M) as three fixed factors.

| **Tests of Between-Subjects Effects** | | | | | |
| --- | --- | --- | --- | --- | --- |
| Dependent Variable: GY | |  |  |  |  |
| Source | Type III Sum of Squares | df | Mean Square | F | Sig. |
| Corrected Model | 3.658E8^a^ | 23 | 1.590E7 | 681.466 | .000 |
| Intercept | 6.121E9 | 1 | 6.121E9 | 2.623E5 | .000 |
| Y | 30033.375 | 1 | 30033.375 | 1.287 | .260 |
| M | 616321.500 | 1 | 616321.500 | 26.411 | .000 |
| N | 3.637E8 | 5 | 7.274E7 | 3.117E3 | .000 |
| Y * M | 91884.375 | 1 | 91884.375 | 3.937 | .051 |
| Y * N | 388449.875 | 5 | 77689.975 | 3.329 | .009 |
| M * N | 813784.500 | 5 | 162756.900 | 6.975 | .000 |
| Y * M * N | 99650.125 | 5 | 19930.025 | .854 | .516 |
| Error | 1680184.000 | 72 | 23335.889 |  |  |
| Total | 6.488E9 | 96 |  |  |  |
| Corrected Total | 3.674E8 | 95 |  |  |  |
| a. R Squared = .995 (Adjusted R Squared = .994) | | | |  |  |

Supplementary Table S8. Analysis of variance summary for water use efficiency (WUE) as affected by cropping year (Y), nitrogen fertilizer levels (N) and plastic mulch (M) as three fixed factors.

| **Tests of Between-Subjects Effects** | | | | | |
| --- | --- | --- | --- | --- | --- |
| Dependent Variable: WUE | |  |  |  |  |
| Source | Type III Sum of Squares | df | Mean Square | F | Sig. |
| Corrected Model | 15.824^a^ | 23 | .688 | 587.148 | .000 |
| Intercept | 306.716 | 1 | 306.716 | 2.617E5 | .000 |
| Y | .003 | 1 | .003 | 2.444 | .122 |
| M | .069 | 1 | .069 | 58.838 | .000 |
| N | 15.611 | 5 | 3.122 | 2.664E3 | .000 |
| Y * M | .006 | 1 | .006 | 5.287 | .024 |
| Y * N | .083 | 5 | .017 | 14.135 | .000 |
| M * N | .047 | 5 | .009 | 8.105 | .000 |
| Y * M * N | .005 | 5 | .001 | .923 | .471 |
| Error | .084 | 72 | .001 |  |  |
| Total | 322.625 | 96 |  |  |  |
| Corrected Total | 15.909 | 95 |  |  |  |
| a. R Squared = .995 (Adjusted R Squared = .993) | | | |  |  |

Supplementary Table S9. Analysis of variance summary for rainfall use efficiency (RUE) as affected by cropping year (Y), nitrogen fertilizer levels (N) and plastic mulch (M) as three fixed factors.

| **Tests of Between-Subjects Effects** | | | | | |
| --- | --- | --- | --- | --- | --- |
| Dependent Variable: RUE | |  |  |  |  |
| Source | Type III Sum of Squares | df | Mean Square | F | Sig. |
| Corrected Model | 10.432^a^ | 23 | .454 | 677.171 | .000 |
| Intercept | 170.978 | 1 | 170.978 | 2.553E5 | .000 |
| Y | .184 | 1 | .184 | 275.397 | .000 |
| M | .018 | 1 | .018 | 26.409 | .000 |
| N | 10.170 | 5 | 2.034 | 3.037E3 | .000 |
| Y * M | .003 | 1 | .003 | 4.560 | .036 |
| Y * N | .031 | 5 | .006 | 9.368 | .000 |
| M * N | .023 | 5 | .005 | 6.757 | .000 |
| Y * M * N | .003 | 5 | .001 | .807 | .548 |
| Error | .048 | 72 | .001 |  |  |
| Total | 181.459 | 96 |  |  |  |
| Corrected Total | 10.480 | 95 |  |  |  |
| a. R Squared = .995 (Adjusted R Squared = .994) | | | |  |  |

Supplementary Table S10. Analysis of variance summary for nitrogen assimilating amount after SS stage (NAAS) as affected by cropping year (Y), nitrogen fertilizer levels (N) and plastic mulch (M) as three fixed factors.

| **Tests of Between-Subjects Effects** | | | | | |
| --- | --- | --- | --- | --- | --- |
| Dependent Variable: NAAS | |  |  |  |  |
| Source | Type III Sum of Squares | df | Mean Square | F | Sig. |
| Corrected Model | 15050.072^a^ | 23 | 654.351 | 109.361 | .000 |
| Intercept | 356295.494 | 1 | 356295.494 | 5.955E4 | .000 |
| Y | 55.313 | 1 | 55.313 | 9.244 | .003 |
| M | 10.514 | 1 | 10.514 | 1.757 | .189 |
| N | 13831.664 | 5 | 2766.333 | 462.334 | .000 |
| Y * M | .723 | 1 | .723 | .121 | .729 |
| Y * N | 811.797 | 5 | 162.359 | 27.135 | .000 |
| M * N | 218.029 | 5 | 43.606 | 7.288 | .000 |
| Y * M * N | 122.033 | 5 | 24.407 | 4.079 | .003 |
| Error | 430.805 | 72 | 5.983 |  |  |
| Total | 371776.371 | 96 |  |  |  |
| Corrected Total | 15480.878 | 95 |  |  |  |
| a. R Squared = .972 (Adjusted R Squared = .963) | | | |  |  |

Supplementary Table S11. Analysis of variance summary for nitrogen harvest index (NHI) as affected by cropping year (Y), nitrogen fertilizer levels (N) and plastic mulch (M) as three fixed factors.

| **Tests of Between-Subjects Effects** | | | | | |
| --- | --- | --- | --- | --- | --- |
| Dependent Variable: NHI | |  |  |  |  |
| Source | Type III Sum of Squares | df | Mean Square | F | Sig. |
| Corrected Model | 1977.541^a^ | 23 | 85.980 | 30.259 | .000 |
| Intercept | 429944.957 | 1 | 429944.957 | 1.513E5 | .000 |
| Y | 2.814 | 1 | 2.814 | .990 | .323 |
| M | 614.291 | 1 | 614.291 | 216.184 | .000 |
| N | 897.257 | 5 | 179.451 | 63.153 | .000 |
| Y * M | 35.600 | 1 | 35.600 | 12.529 | .001 |
| Y * N | 194.139 | 5 | 38.828 | 13.664 | .000 |
| M * N | 190.052 | 5 | 38.010 | 13.377 | .000 |
| Y * M * N | 43.388 | 5 | 8.678 | 3.054 | .015 |
| Error | 204.589 | 72 | 2.842 |  |  |
| Total | 432127.086 | 96 |  |  |  |
| Corrected Total | 2182.130 | 95 |  |  |  |
| a. R Squared = .906 (Adjusted R Squared = .876) | | | |  |  |

Supplementary Table S12. Analysis of variance summary for nitrogen use efficiency (NUE) as affected by cropping year (Y), nitrogen fertilizer levels (N) and plastic mulch (M) as three fixed factors.

| **Tests of Between-Subjects Effects** | | | | | |
| --- | --- | --- | --- | --- | --- |
| Dependent Variable: NUE | |  |  |  |  |
| Source | Type III Sum of Squares | df | Mean Square | F | Sig. |
| Corrected Model | 32492.403^a^ | 19 | 1710.126 | 1.774E3 | .000 |
| Intercept | 151457.435 | 1 | 151457.435 | 1.571E5 | .000 |
| Y | 9.060 | 1 | 9.060 | 9.398 | .003 |
| M | 39.881 | 1 | 39.881 | 41.367 | .000 |
| N | 32384.761 | 4 | 8096.190 | 8.398E3 | .000 |
| Y * M | .378 | 1 | .378 | .392 | .534 |
| Y * N | 29.101 | 4 | 7.275 | 7.546 | .000 |
| M * N | 25.833 | 4 | 6.458 | 6.699 | .000 |
| Y * M * N | 3.389 | 4 | .847 | .879 | .482 |
| Error | 57.845 | 60 | .964 |  |  |
| Total | 184007.682 | 80 |  |  |  |
| Corrected Total | 32550.247 | 79 |  |  |  |
| a. R Squared = .998 (Adjusted R Squared = .998) | | | |  |  |

Supplementary Table S13. Analysis of variance summary for nitrogen apparent recovery efficiency (NRE) as affected by cropping year (Y), nitrogen fertilizer levels (N) and plastic mulch (M) as three fixed factors.

| **Tests of Between-Subjects Effects** | | | | | |
| --- | --- | --- | --- | --- | --- |
| Dependent Variable: NRE | |  |  |  |  |
| Source | Type III Sum of Squares | df | Mean Square | F | Sig. |
| Corrected Model | 8651.507^a^ | 19 | 455.342 | 193.213 | .000 |
| Intercept | 90629.911 | 1 | 90629.911 | 3.846E4 | .000 |
| Y | .829 | 1 | .829 | .352 | .555 |
| M | 9.034 | 1 | 9.034 | 3.833 | .055 |
| N | 8481.563 | 4 | 2120.391 | 899.734 | .000 |
| Y * M | 7.503 | 1 | 7.503 | 3.184 | .079 |
| Y * N | 132.180 | 4 | 33.045 | 14.022 | .000 |
| M * N | 16.480 | 4 | 4.120 | 1.748 | .151 |
| Y * M * N | 3.917 | 4 | .979 | .416 | .797 |
| Error | 141.401 | 60 | 2.357 |  |  |
| Total | 99422.819 | 80 |  |  |  |
| Corrected Total | 8792.908 | 79 |  |  |  |
| a. R Squared = .984 (Adjusted R Squared = .979) | | | |  |  |

Supplementary Table S14. Analysis of variance summary for partial factor productivity of the fertilizer (PFP) as affected by cropping year (Y), nitrogen fertilizer levels (N) and plastic mulch (M) as three fixed factors.

| **Tests of Between-Subjects Effects** | | | | | |
| --- | --- | --- | --- | --- | --- |
| Dependent Variable: PFP | |  |  |  |  |
| Source | Type III Sum of Squares | df | Mean Square | F | Sig. |
| Corrected Model | 1179.014^a^ | 19 | 62.053 | 355.428 | .000 |
| Intercept | 38681.037 | 1 | 38681.037 | 2.216E5 | .000 |
| Y | 1.028 | 1 | 1.028 | 5.886 | .018 |
| M | 8.545 | 1 | 8.545 | 48.947 | .000 |
| N | 1162.304 | 4 | 290.576 | 1.664E3 | .000 |
| Y * M | .277 | 1 | .277 | 1.584 | .213 |
| Y * N | 4.073 | 4 | 1.018 | 5.832 | .000 |
| M * N | 2.030 | 4 | .507 | 2.907 | .029 |
| Y * M * N | .757 | 4 | .189 | 1.083 | .373 |
| Error | 10.475 | 60 | .175 |  |  |
| Total | 39870.526 | 80 |  |  |  |
| Corrected Total | 1189.489 | 79 |  |  |  |
| a. R Squared = .991 (Adjusted R Squared = .988) | | | |  |  |
